# Terminal Axonal Arborization and Synaptic Bouton Formation Critically Rely on Abp1 and the Arp2/3 Complex

**DOI:** 10.1371/journal.pone.0097692

**Published:** 2014-05-19

**Authors:** Nicole Koch, Oliver Kobler, Ulrich Thomas, Britta Qualmann, Michael M. Kessels

**Affiliations:** 1 Institute for Biochemistry I, Jena University Hospital - Friedrich Schiller University Jena, Jena, Germany; 2 Research Group Membrane Trafficking and Cytoskeleton, Department of Neurochemistry & Molecular Biology, Leibniz Institute for Neurobiology, Magdeburg, Germany; 3 Research Group Functional Genetics of the Synapse, Department of Neurochemistry & Molecular Biology, Leibniz Institute for Neurobiology, Magdeburg, Germany; Biogen Idec, United States of America

## Abstract

Neuronal network formation depends on properly timed and localized generation of presynaptic as well as postsynaptic structures. Although of utmost importance for understanding development and plasticity of the nervous system and neurodegenerative diseases, the molecular mechanisms that ensure the fine-control needed for coordinated establishment of pre- and postsynapses are still largely unknown. We show that the F-actin-binding protein Abp1 is prominently expressed in the *Drosophila* nervous system and reveal that Abp1 is an important regulator in shaping glutamatergic neuromuscular junctions (NMJs) of flies. STED microscopy shows that Abp1 accumulations can be found in close proximity of synaptic vesicles and at the cell cortex in nerve terminals. *Abp1* knock-out larvae have locomotion defects and underdeveloped NMJs that are characterized by a reduced number of both type Ib synaptic boutons and branches of motornerve terminals. Abp1 is able to indirectly trigger Arp2/3 complex-mediated actin nucleation and interacts with both WASP and Scar. Consistently, Arp2 and Arp3 loss-of-function also resulted in impairments of bouton formation and arborization at NMJs, i.e. fully phenocopied *abp1* knock-out. Interestingly, neuron- and muscle-specific rescue experiments revealed that synaptic bouton formation critically depends on presynaptic Abp1, whereas the NMJ branching defects can be compensated for by restoring Abp1 functions at either side. In line with this presynaptic importance of Abp1, also presynaptic Arp2 and Arp3 are crucial for the formation of type Ib synaptic boutons. Interestingly, presynaptic Abp1 functions in NMJ formation were fully dependent on the Arp2/3 complex, as revealed by suppression of Abp1-induced synaptic bouton formation and branching of axon terminals upon presynaptic Arp2 RNAi. These data reveal that Abp1 and Arp2/3 complex-mediated actin cytoskeletal dynamics drive both synaptic bouton formation and NMJ branching. Our data furthermore shed light on an intense bidirectional functional crosstalk between pre- and postsynapses during the development of synaptic contacts.

## Introduction

The establishment of functional neuronal circuits requires that neurons develop axons and dendritic trees and that synapses are formed at specific locations. This involves a plethora of molecular components to mediate a coordinated formation of pre- and postsynapses. Various growth-promoting signals have been implicated in the regulation of neuromorphogenesis, synapse formation and synaptic plasticity. Studies on the *Drosophila melanogaster* larval neuromuscular junction (NMJ), a powerful model system for glutamatergic synapses, have uncovered signaling mechanisms orchestrating the growth of pre- and postsynaptic structures [1]–[2]. Relatively little, however, is known about the effectors that actually bring about the changes in neuronal morphology and organization that give rise to properly developed NMJs.

While the importance of the microtubular cytoskeleton for NMJ expansion is well established [3], a putative role of the actin cytoskeleton is less clear. On one side, analyses of flies deficient for *wasp*, an activator of the actin nucleation machinery Arp2/3 complex, displayed enlarged NMJs [4]–[5] - an observation that seems to suggest that F-actin formation has a negative impact on synapse formation. On the other side, the small GTPase Rac has been shown to have a promoting role in axonal branching in mushroom body neurons of *Drosophila* brains [6] and retrograde BMP signaling, a major growth promoting pathway at larval NMJs, induced motoneuronal expression of the Rac GEF Trio [7]. Also the actin filament promoting formin Diaphanous seems to be important in bouton formation, presumably acting via interactions with both F-actin and microtubules [8] and the Ena/VASP-binding and actin filament growth-promoting protein lamellipodin (MIG-10) [9] was identified as critical for NMJ formation in *C. elegans* AIY interneurons [10]. Finally, PI3K signaling, which could also include interactions of the PI3K product phosphatidyl-inositol-(3,4,5)-trisphosphate (PIP_3_) with actin-binding proteins, promoted both NMJ expansion [11] and terminal axon branching in *Drosophila* [12]–[13]. It thus seemed possible that terminal arborization and presynapse formation employ shared molecular mechanisms and that these involve actin dynamics-promoting factors.

Here we show that knock-out of *abp1* in *D. melanogaster* leads to locomotion defects along with both impaired synaptic bouton formation and defective axon terminal arborization at larval NMJs. Abp1 is an F-actin-binding protein and also associates with lipids including PIP_3_. Abp1 associates with dynamic F-actin structures in response to Rac1 activation and is able to mediate the formation of new actin filaments by interfacing with actin nucleation machineries or with their activator proteins [14]–[18]. Interestingly, defects in NMJ development and larval migration observed in *abp1* knock-out flies were fully phenocopied by depletion of Arp2 or Arp3, integral components of the Arp2/3 complex actin nucleation machinery. Our studies reveal that presynaptic functions of Abp1 in the NMJ require presynaptic Arp2/3 complex and that Abp1 associates with both WASP and Scar in the fly nervous system. The identification of the Arp2/3 complex and Abp1 as crucial components in NMJ formation suggests that Abp1 and Arp2/3 complex-mediated promotion of actin filament formation are shared molecular key requirements underlying both synaptic bouton formation and axon terminal arborization.

## Material and Methods

### Fly strains

Fly strains used are summarized in table 1 and 2. All crosses were performed at 25°C unless indicated otherwise. *w^1118^* served as a control (*wt*).

**Table 1:** Mutant and transgenic alleles used.

Mutant and transgenic alleles	Alternative (short) name	Source/reference
*abp1* ^KO^		[15]
*abp1* ^WH-rev^		[15]
UAS-Abp1 RNAi	Abp1 RNAi	[15]
UAS-Abp1-GFP	Abp1-GFP	
*w* ^1118^		Bloomington Drosophila Stock Center
PBac insertion line WH f05024	*abp1* ^WH f05024^	Exelixis collection
PBac insertion line *Df(3L)Exel6119*	Df(3L)	Exelixis collection
UAS-Arp2 RNAi #1 (VDRC 29643)	Arp2 RNAi #1	[28]
UAS-Arp2 RNAi #2 (VDRC 29644)	Arp2 RNAi #2	Vienna Drosophila *RNAi* Center
UAS-Arp3 RNAi (VDRC 35260)	Arp3 RNAi	[46]
*wasp* ^1^		[47]
*wasp* ^3^		[47]
*scar* ^k03107/+^		[48]
*scar^Δ^* ^37/+^		[41]–[42]
UAS-Scar RNAi (VDRC 21908)	Scar RNAi	[49]
UAS-WASP RNAi	WASP RNAi	Vienna Drosophila *RNAi* Center
Gal4-activator strain C57*-Gal4*		[50]
Gal4-activator strain OK371-*Gal4*		[51]
Gal4-activator strain Ubi-*Gal4*		[52]

**Table 2:** Genotypes generated for knock-down, overexpression and rescue experiments.

genotypes	Alternative (short) name
UAS*-Abp1/+*; C57*-Gal4 abp1^KO^/Df(3L)*	C57-*Gal4*+Abp1+*abp1* ^KO^/Df(3L)
OK371*-Gal4/*UAS*-Abp1; abp1^KO^/Df(3L)*	OK371-*Gal4*+Abp1+*abp1* ^KO^/Df(3L)
Ubi-*Gal4/+*; UAS*-Arp2 RNAi #1/+*	Ubi-*Gal4*+Arp2 RNAi #1
Ubi-*Gal4/+*; UAS*-Arp2 RNAi #2/+*	Ubi-*Gal4*+Arp2 RNAi #2
Ubi*-Gal4/+*; UAS*-Arp3 RNAi/+*	Ubi-*Gal4*+Arp3 RNAi
OK371*-Gal4/+*; UAS-*Abp1 RNAi/+*	OK371-*Gal4*+Abp1 RNAi
OK371*-Gal4/+*; UAS-*Arp3 RNAi/+*	OK371-*Gal4*+Arp3 RNAi
OK371*-Gal4/+*; UAS-*Arp2 RNAi #2/+*	OK371-*Gal4*+Arp2 RNAi #2
OK371*-Gal4/+*; UAS-*Arp3 RNAi/+*	OK371-*Gal4*+Arp3 RNAi
OK371*-Gal4/*UAS-*Abp1-GFP*	OK371-*Gal4*+Abp1-GFP
OK371*-Gal4/*UAS*-Abp1-GFP*; UAS*-Arp2 RNAi #2/+*	OK371-*Gal4*+Abp1-GFP+Arp2 RNAi #2
*wasp^3^ abp1^KO^/wasp^1^- Df(3L)*	*wasp* ^1/3^+*abp1^KO^/Df(3L)*
*scar* ^k03107^ *abp1^KO^/Df(3L)*	*scar* ^k03107/+^+*abp1^KO^/Df(3L)*
OK371*-Gal4/+*; UAS-*WASP RNAi/+*	OK371-*Gal4*+WASP RNAi
OK371*-Gal4/+*; UAS-*Scar RNAi/+*	OK371-*Gal4*+Scar RNAi
C57*-Gal4/*UAS*-WASP RNAi*	C57-*Gal4*+WASP RNAi
C57*-Gal4/*UAS*-Scar RNAi*	C57-*Gal4*+Scar RNAi

### DNA constructs and recombinant proteins

Abp1 RNAi [15] and Abp1-GFP were cloned into pUAST. Abp1 SH3^P523A^ (Abp1 SH3*) [15] as well as the SH3 domain of Abp1 were cloned into pGEX-5X-1.

Bacterial fusion proteins were expressed and purified according to Kessels and Qualmann [19].

### Antibodies, sample preparation and immunofluorescence analyses

Affinity-purified anti-Abp1 antibodies described previously were used at 1∶100 for immunostainings [15]. Other primary antibodies used include rabbit anti-GFP (1∶1000; Invitrogen and Abcam, respectively), guinea pig anti-WASP (1∶250) and anti-Scar (1∶250) [20]. The mouse monoclonal antibodies against Dlg (4F3; 1∶500), FasciclinII (FasII) (1D4; 1∶10), Futsch (22C10; 1∶10), Bruchpilot (BRP) (NC82; 1∶100) and CSP (DCSP3; 1∶50) all were from the Developmental Studies Hybridoma Bank (University of Iowa). Goat anti-HRP-Cy5 conjugates were from Jackson Laboratories.

Immunostaining of whole mount embryos was done according to Goldstein and Fyrberg [21]. Body wall preparations of 3^rd^ instar larvae were performed as described [22].

Primary antibody incubations were performed overnight at 4°C or for 2 hours at RT. Fluorescently-labeled HRP (Jackson laboratories) was co-incubated with fluorophore-conjugated secondary antibodies for 1 hour at RT. Secondary antibodies used at 1∶200 were, anti-mouseAlexa-568, anti-mouse-Cy3, anti-guinea pigAlexa-488, anti-rabbit-Alexa488 (Molecular Probes) and anti-rabbit-Atto647N (Sigma) and anti-mouse-Abberior STAR580 (Abberior GmbH) for STED-microscopy.

Confocal imaging was performed on a Leica TCS SP2, a Zeiss CellObserver with Apotome or a Zeiss CellObserverSD. In some cases, confocal images were deconvolved with Huygens Professional by applying default values to the *Deconvolution wizard*. The *Quality threshold* was set to 0.5. Samples designated for direct comparison were analyzed in parallel using identical settings.

HRP-conjugated secondary antibodies for Western blotting were from Dianova.

### STED microscopy

STED stacks were acquired on a Leica TCS SP5 2-channel STED microscope equipped with an inverted microscope DMI 6000 and a 100x STED objective (HCX PL APO 100x, 1.4 NA oil STED, all parts are from Leica Microsystems). Abberior STAR580 and Atto647N were sequentially excited using pulsed-diode lasers (PicoQuant) at 531 nm and 635 nm and fluorescence signals were detected with avalanche photo diodes (Perkin Elmer Inc.) through BL HC 607/36 and ET BP 670/30 emission filters (AHF Analysentechnik AG) separated by a dichroic beam splitter at 650 nm, respectively. Depletions were done at 730 nm for STAR580 and at 750 nm for Atto647N with a Titanium sapphire laser (Chameleon ultra II, Coherent). Applying a Zoom of 6 at 1024×1024 and using 125.8 nm z-steps (system optimized) results in a pixel size of 25.2 nm. The scan speed was set to 700 Hz by applying 48x line averaging.

All acquired STED stacks were subsequently deconvolved using the STED package Huygens Professional (SVI, v 4.4.0p9) as follows: Beside the optical microscopic parameters provided by the lif-file itself, default pulsed STED parameters, values recommended by SVI, were applied to the build-in *Parameter editor* to calculate a theoretical point spread function (PSF) specific for the Ti:Sapphire-STED system. Within the *Deconvolution wizard*, stacks were first subjected to a specific *STED thermal drift correction* algorithm (*Stabilization*) to ensure proper calculating of the PSF within oversampled STED stacks. Subsequent cropping and automatic background calculation were applied. For deconvolution, the *Signal to noise ratios* were set to 10 for both channels. The *Optimized iteration mode* was applied until it reached a *Quality threshold* of 0.01. Subsequently, the *Chromatic shift wizard* was applied to equalize the chromatic shift between both channels in depth.

### Quantitative analysis of NMJ morphology and of the distribution of immunolabeled, endogenous Abp1 at NMJs

For each genotype, type Ib boutons and nerve terminal branch points were examined using maximum projections of confocal stacks of muscle 6/7 (segment A2) from 18–42 NMJs (10–23 larvae). Anti-Dlg and anti-HRP immunostaining allowed for discrimination between type Ib and type Is boutons. For nerve terminal branching, only axonal branches with 2 or more type Ib boutons were analyzed. Statistical analyses were performed using two-tailed Student's *t* test and One Way ANOVA post Tukey analysis, respectively.

Abp1 localization at NMJs in relation to localizations of synaptic markers was analyzed using maximum intensity projections of z-stacks and the *Plot Profil*e tool of ImageJ.

### Pull-down assays

Fly heads were harvested by agitating approximately 50 shock-frozen flies per sample in a sieve and homogenates were obtained by incubation for 45 min at 4°C in 0.5 ml HEPES buffer (10 mM HEPES pH 7.4, 1 mM EGTA, 0.1 mM MgCl_2_, 150 mM NaCl, Roche Complete protease inhibitor) containing 1% Triton X-100 and centrifugation at 16,000xg for 10 min. The supernatants were then used for pull-down assays, as described in Qualmann et al. [23].

### Larval migration assay

3^rd^ instar larvae were transferred to an apple juice-agar plate overlaying a 5 mm-grid. Upon migration onset, migration tracks of larvae were recorded and plotted. The analyses were done at the same time of the day and under constant environmental conditions. 17–38 larvae/genotype were examined. Statistical analyses were performed using the two-tailed Student's *t* test and One Way ANOVA post Tukey analysis, respectively.

## Results

### Abp1 knock-out larvae show locomotion defects

**Figure 1:**
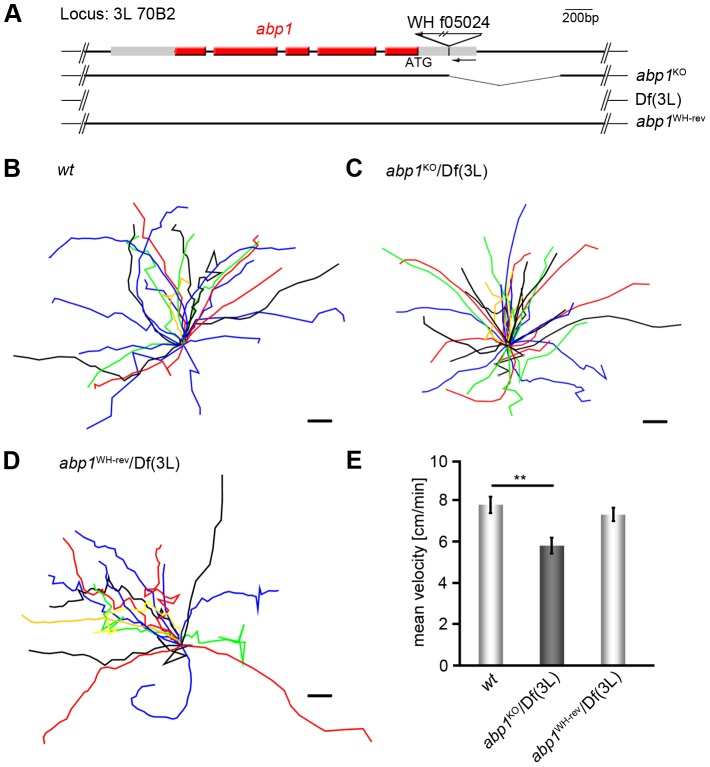
*Abp1* knock-out leads to impaired larval migration. (**A**) Genomic scheme of *abp1* (*CG10083*) locus 3 L 70B2 with PBac insertion WH f05024, of *abp1* knock-out (*abp1* ^KO^) *flies* generated by FRT-mediated recombination of PBac insertions, of the deficiency Df(3 L) and of the revertant *abp1* ^WH-rev^. Shown are exons with translated (red) and untranslated regions (grey). (**B–D**) Summarized presentation of migration tracks with centered common start points (bars, 5 mm) of *wt* (**B**), *abp1* ^KO^/Df(3 L) (**C**) and *abp1* ^WH-rev^/Df(3 L) (**D**). (**E**) Mean velocities of larval migration (n = 17–38). Data represent mean±SEM. ** = p<0.01. One way ANOVA post Tukey.

In mammals, the F-actin-binding protein Abp1 is highly expressed in the nervous system and has been implicated in actin dynamics, as it associates with newly forming actin filaments and interfaces with different actin nucleation machineries, among those the Arp2/3 complex activator Neural Wiskott-Aldrich Syndrome protein (N-WASP) [18] and Cobl [16]. In flies, Abp1 interacts with the Arp2/3 complex activator Scar and *abp1* knock-out flies (for genomic scheme see Fig. 1A) show defects in the development of micro- and macrochaete bristles reflecting impairments in the otherwise highly regular F-actin organization of these sensory organs at the thorax of flies [15].

In order to obtain general insights into neuronal functions of Abp1 in higher eukaryotes, we monitored the locomotor behavior of *abp1* knock-out larvae. The *abp1* mutants appeared less active than *wt* larvae. Paralysis, however, was not observed and recording the tracks of migration did not reveal any obvious differences in locomotion patterns (Fig. 1B,C). Analyses of travelled distances showed that the migration tracks of larvae lacking *abp1* were significantly shorter than those of *wt* flies recorded for the same time period (Fig. 1B,C). Calculations of the velocities of migration revealed that the average speed of *abp1* knock-out larvae was only 74% of that of *wt* larvae (Fig. 1B,C,E).

Importantly, precise excision of the PBac insertion WH f05024 (*abp1* ^WH-rev^/Df(3 L)) did not only restore Abp1 expression, as demonstrated by anti-Abp1 immunoblotting of body wall and fly head preparations (data not shown; compare Koch et al., 2012), but also led to normal larval migration indistinguishable from *wt* (Fig. 1D,E). These evaluations demonstrated that the larval migration phenotype observed for *abp1* ^KO^/Df(3 L) larvae was specifically caused by *abp1* disruption.

### Abp1 is highly expressed in the nervous system

**Figure 2:**
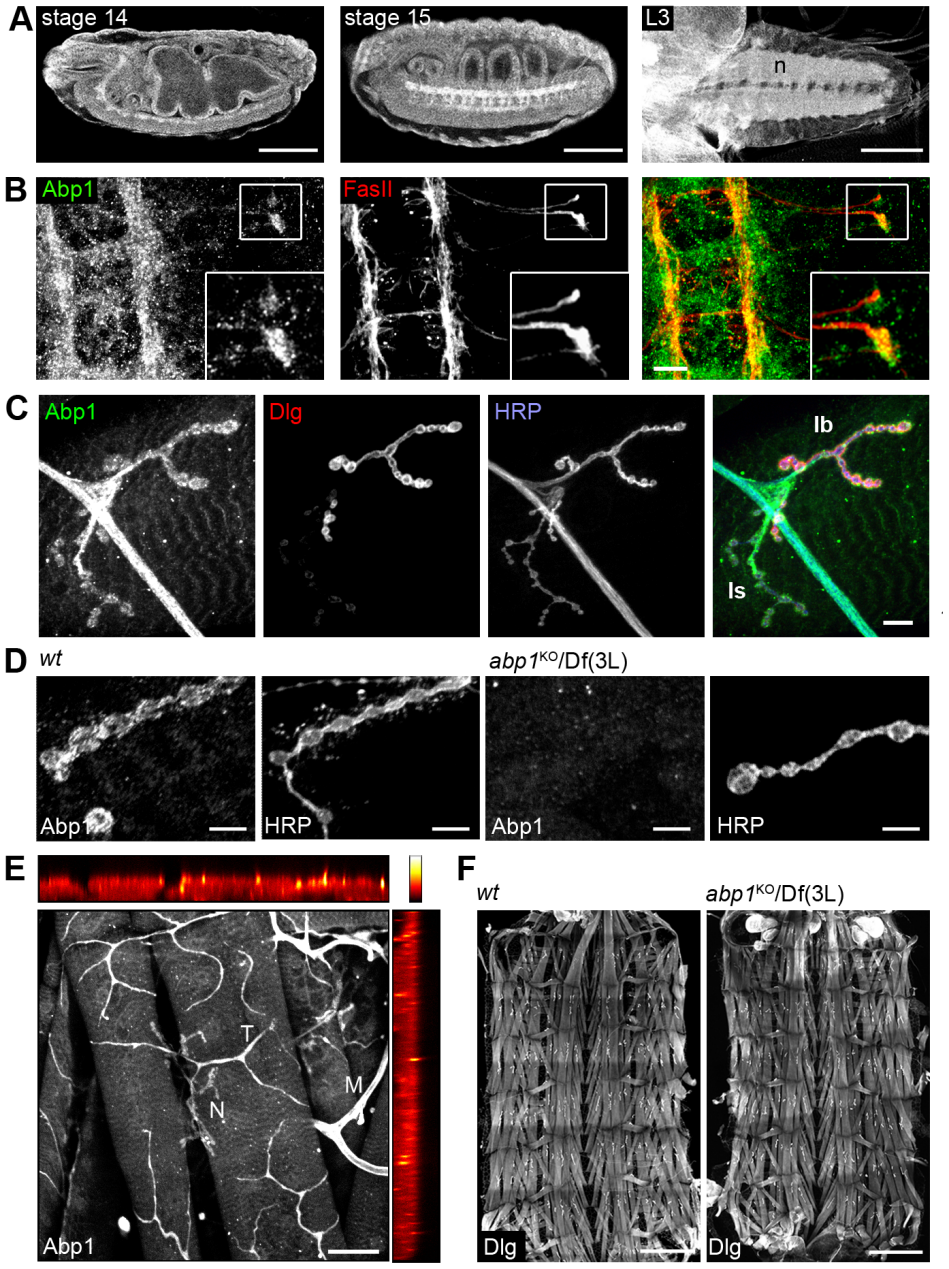
Abp1 is highly expressed in the central nervous system. (**A**) Immunolabeling of endogenous Abp1 at indicated stages of development. Left, confocal lateral view of stage 14 embryo; middle, confocal, ventro-lateral view of stage 15 embryo; right, confocal image of a 3^rd^ instar larval brain. Embryos display strong Abp1 immunoreactivity in epithelia and the CNS (left), in the commissures and longitudinal tracks of the CNS (middle) as well as in the neuropil (n) of the ventral nerve cord of 3^rd^ instar larvae. Bars, 100 µm. (**B**) Maximum projections of a confocal stack of a stage 15 embryo immunostained with anti-Abp1 antibodies showing that besides in the commissures and longitudinal tracks of the CNS Abp1 is present in motoneuronal growth cones marked with anti-FasII antibodies. Boxed area is presented as enlarged inset. Bar, 10 µm. (**C**) Abp1 immunoreactivity at NMJs of a 3^rd^ instar larvae costained with postsynaptic (Dlg) and presynaptic markers (HRP). Note that in contrast to Dlg labeling, Abp1 immunoreactivity is nearly equally strong at type Ib and type Is synaptic boutons. Bar, 10 µm. (**D**) Anti-Abp1 immunostaining of *wt* versus *abp1^KO^*/Df(3 L) NMJs showing that the anti-Abp1 immunosignal at NMJs is specific. Bar, 10 µm. (**E**) Overview of anti-Abp1 immunostaining at muscles 6/7 NMJs of a 3^rd^ instar larvae (maximum projections of confocal stack). Note that besides a low expression in muscles, Abp1 is enriched in tracheae (example marked with T), axons of motoneurons (example marked with M) and NMJs (example marked by N). Colored panels at top and right display the intensity of the anti-Abp1 signal in Z-direction in pseudo colors encoding for the fluorescence intensity (see scale at upper right). Bar, 50 µm. (**F**) Maximum projections of stitched confocal images of anti-Dlg-stained body wall preparations show normal segmentation, muscle organization and innervations of an *wt* (left) and an *abp1* ^KO^/Df(3 L) fly (right). Bar, 500 µm.

Immunofluorescence microscopy analyses on whole-mount embryos revealed that Abp1 is widely expressed throughout embryogenesis. Abp1 expression was particularly prominent in both the embryonic and the larval CNS (Fig. 2A). Examinations of stage 15 embryos at higher magnification showed that Abp1 was detectable in motoneuronal growth cones marked with antibodies against the cell adhesion molecule FasciclinII (FasII) (Fig. 2B). This finding suggested a potential role of Abp1 in developmental processes leading to the formation of NMJs.

We therefore more closely analyzed the localization of Abp1 at developmental stages marked by established NMJs. In 3^rd^ instar larvae, Abp1 was prominently immunolabeled in both the axons of motoneurons and in NMJs, as shown by colocalization with anti-HRP staining highlighting axonal and presynaptic membranes. Counterstaining against the postsynaptic marker Dlg showed that Abp1 was localized to presynaptic and/or postsynaptic sides of Dlg-enriched type Ib boutons and to type Is boutons (Fig. 2C).

Control incubations on *abp1* ^KO^/Df(3 L) larvae demonstrated that the anti-Abp1 immunolabeling at NMJs was specific. No anti-Abp1 immunosignal was obtained when *abp1* was knocked out (Fig. 2D).

In addition to Abp1's expression in the central nervous system, in motoneurons and its presence at NMJs (Fig. 2C–E), Abp1 was also enriched in tracheae and some Abp1 was furthermore found at the cortex of muscle cells (Fig. 2E).

Theoretically, the migration defects of *abp1* knock-out larvae (Fig. 1) may reflect defects of sensory input, information processing in the brain, motoneuronal defects and/or muscle defects. Examinations of the muscles did not reveal any size differences between muscles from *abp1* ^KO^/Df(3L) and *wt* flies (our unpublished results). Likewise, no overt defects of overall muscle patterning were observed in *abp1* ^KO^/Df(3 L) animals (Fig. 2F). In the following we thus concentrated on Abp1's functions at NMJs.

### Abp1 is localized both pre- and postsynaptically

**Figure 3:**
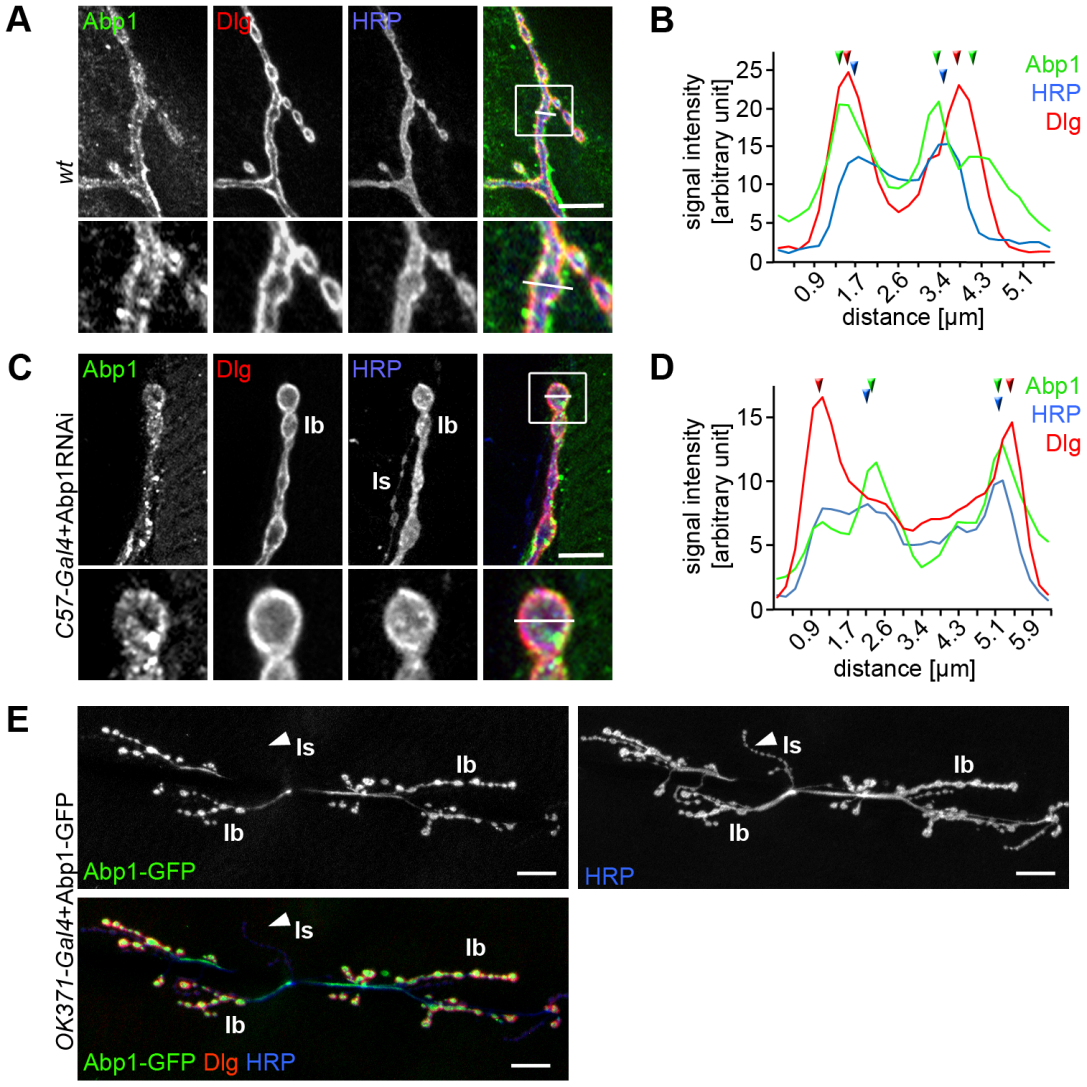
Abp1 is present on both the presynaptic and the postsynaptic side of NMJs. (**A–D**) Localization studies of Abp1 in HRP and anti-Dlg-stained *wt* NMJs (**A,B**) compared to anti-Abp1 immunolabeling in NMJs of flies expressing C57-induced postsynaptic Abp1 RNAi demonstrating the less obvious presynaptic pool of Abp1 (**C,D**). Lower panels represent enlargements of boxed areas. Note that presynaptic endogenous Abp1 immunolabeling was not detectable in type Is boutons but is enriched in type Ib boutons (**C**). (**B,D**) Quantitative analyses of fluorescence intensity plots across type Ib boutons along the lines shown in **A** and **C** allow for resolving spatially distinct fluorescence maxima for Dlg and HRP (marked by arrow heads in **B** and **D**). (**B**) Anti-Abp1 immunostaining spans the localization of both markers (or even extends postsynaptically) and shows segregated pre- and postsynaptic peaks, respectively. (**D**) Postsynaptic Abp1 RNAi drastically reduces the prominent postsynaptic anti-Abp1 signals and thereby reveals a remaining anti-Abp1 immunosignal overlapping with the presynaptic HRP localization. Bars in **A** and **C**, 10 µm. (**E**) Presynaptically expressed Abp1-GFP at NMJs at muscles 6/7 of abdominal segment A2 is enriched at Dlg-enriched structures, whereas Abp1 localization at interbouton axon segments and type Is boutons is low. Bars, 20 µm.

We first asked whether the presence of Abp1 at NMJs reflected a presynaptic or postsynaptic localization of Abp1 or both. Coimmunolabelings with anti-HRP-labeled presynapses and with antibodies against the postsynaptic marker Dlg (Fig. 3A) followed by fluorescence maxima analyses (Fig. 3B; arrows) strongly suggested that Abp1 is present on both sides of NMJs.

As also suggested from the overview analyses (Fig. 2E), anti-Abp1 immunodetection hereby often appeared more postsynaptic, as it usually extended with lower intensities even beyond the Dlg labeling (Fig. 3A,B).

The postsynaptic Abp1 labeling might obscure presynaptic labeling. In order to explicitly visualize the presynaptic subpool of Abp1 suggested from the observation that also axons were highlighted by anti-Abp1 immunolabeling (Fig. 2C,E), we knocked down Abp1 postsynaptically using UAS-Abp1 RNAi and the muscle-specific *Gal4*-activator C57. We then evaluated the remaining anti-Abp1 immunostaining in parallel to that of HRP and Dlg. As expected, the anti-Abp1 immunosignal with fluorescence maxima spatially overlapping with those of Dlg was markedly reduced upon postsynaptic Abp1 RNAi. Yet, despite the postsynaptic knockdown of Abp1, a significant portion of anti-Abp1 immunolabeling remained and overlapped with the anti-HRP signal (Fig. 3C,D). These experiments thus clearly revealed an additional, presynaptic pool of Abp1 overlapping with HRP.

From the fact that both type Ib and type Is boutons were labelled in *wt* flies (Fig. 2C) but upon postsynaptic Abp1 RNAi only type Ib synaptic boutons retained detectable anti-Abp1 immunostaining, it can be concluded that, in the muscle cells, Abp1 is enriched at postsynaptic structures in contact with both types of boutons, whereas, in motoneurons contacting these muscles, Abp1 preferentially occurs in type Ib boutons.

In order to confirm that presynaptic Abp1 at NMJs indeed is specifically enriched in type Ib boutons and that the data for endogenous Abp1 were not affected by some putative immunohistochemical artifacts, we next visualized the localization of presynaptic Abp1 using a transgenic line expressing Abp1-GFP under the control of the presynaptic driver OK371-*Gal4*. Presynaptically expressed Abp1-GFP displayed similar distribution as revealed for the endogenous anti-Abp1 immunostaining remaining after knock-down of postsynaptic Abp1. Axon segments joining boutons and type Is boutons showed no accumulation of Abp1 whereas type Ib boutons were enriched for Abp1-GFP (Fig. 3E).

### Lack of Abp1 impairs both synaptic bouton formation and axon terminal branching leading to NMJs with reduced complexity

**Figure 4:**
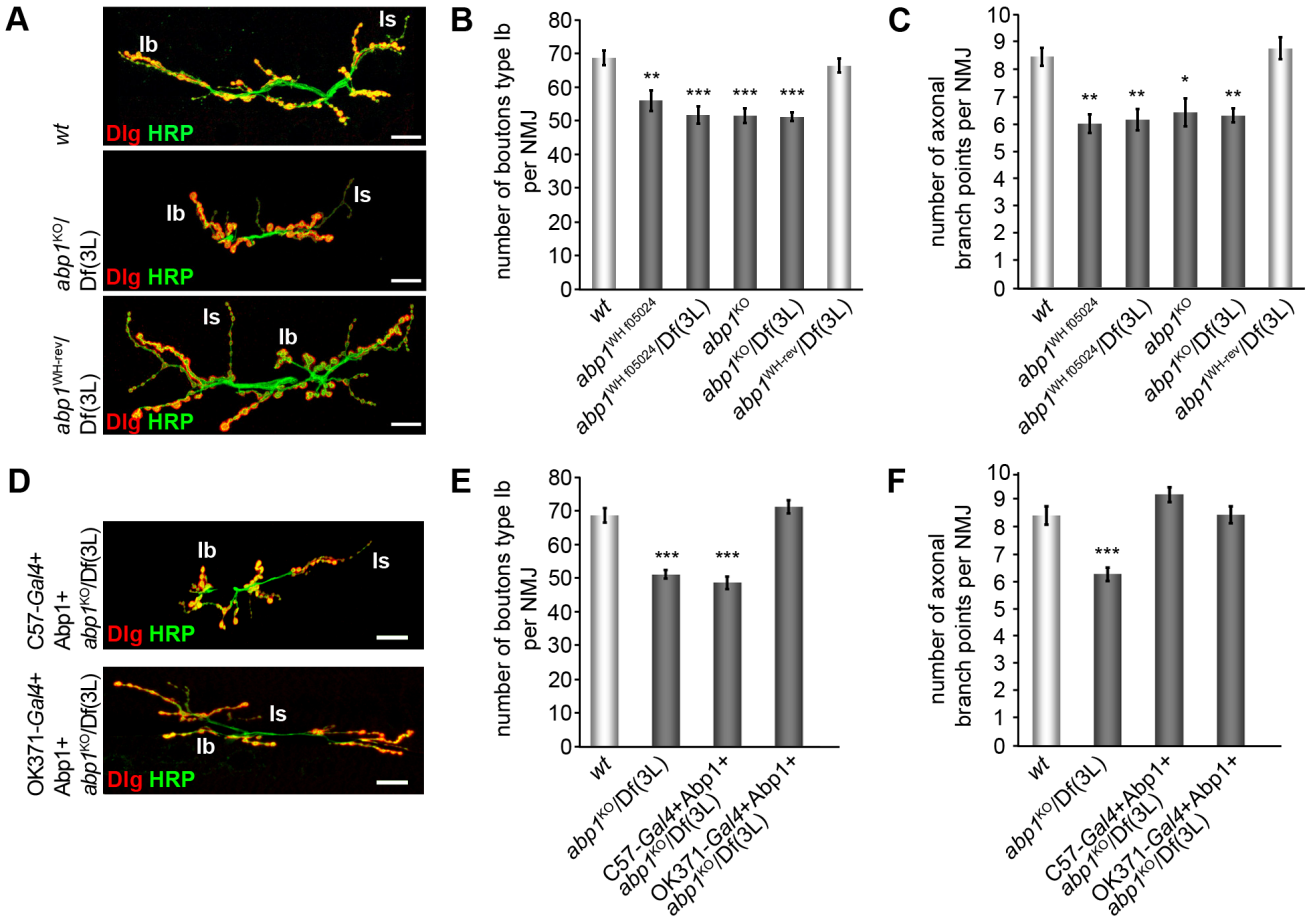
Abp1 deficiency causes defects in NMJ development. (**A**) Examples of NMJs at muscles 6/7 of abdominal segment A2 from *wt*, *abp1* ^KO^/Df(3 L) and hemizygous *abp1^WH-rev^* larvae, respectively, stained for pre- and postsynaptic markers HRP (green) and Dlg (red). Note that the NMJs of *abp1* ^KO^/Df(3 L) flies (middle) appear condensed and less arborized and contain fewer synaptic boutons type Ib when compared to *wt* and to revertants generated to restore *abp1* expression by precise excision to demonstrate the specificity of *abp1* knock-out phenotypes. Bars, 20 µm. (**B,C**) Quantification of type Ib bouton numbers (**B**) and terminal axonal branch points (**C**). The significant reduction in the number of boutons and branch points observed in all *abp1*-mutant allelic combinations are restored in the revertant *abp1^WH-rev^*. (**D**) HRP and anti-Dlg-stained NMJs of *abp1*-deficient larvae with postsynaptic Abp1 re-expression (C57*-Gal4*+Abp1+*abp1* ^KO^/Df(3 L)) and of larvae with a presynaptic rescue of Abp1 functions (OK371-*Gal4*+Abp1+*abp1* ^KO^/Df(3 L)). Bar, 20 µm. (**E,F**) Quantitative analyses of type Ib boutons and of terminal axonal branch points show that only presynaptic expression of Abp1 rescues bouton number whereas the number of terminal axonal branch points can be rescued by both pre- and postsynaptic expression of Abp1. Data represent mean±SEM. n = 19–42 NMJs/genotype. * = p<0.05, ** = p<0.01 and *** = p<0.001. One way ANOVA post Tukey.

Despite the expression of Abp1 in growth cones (Fig. 2B) we neither observed inappropriate routing of motoneuronal axons nor a lack of innervation at individual muscles. Analyses of NMJs, however, showed that NMJ expansion and complexity were significantly reduced in 3^rd^ instar *abp1* knock-out larvae (Fig. 4A). Detailed quantitative analyses of anti-HRP and anti-Dlg-stained NMJs revealed that the number of type Ib boutons as well as the arborization of motornerve endings were both reduced by about 25% at *abp1* ^KO^/Df(3 L) compared to *wt* NMJs (Fig. 4B,C).

Less expanded and less complex NMJs were consistently observed in all genotypes that result in Abp1 deficiency. Similar to NMJs of homozygous *abp1* ^KO^ or *abp1* ^KO^/Df(3 L) larvae, those of a strong hypomorphic allele, *abp1* ^WH f05024^ [15], displayed significant reductions of bouton numbers and of nerve terminal branching in either homozygosity or when placed over the deficiency Df(3L) to minimize putative second site mutational effects (Fig. 4A–C).

Importantly, revertants with precise excision of the PBac insertion WH f05024 (*abp1* ^WH-rev^/Df(3 L)) exhibiting restored Abp1 levels [15] displayed well-elaborated NMJs indistinguishable from *wt* (Fig. 4A–C). These evaluations demonstrated that the phenotypes observed for *abp1* ^KO^, *abp1* ^KO^/Df(3 L), abp1^WH f05024^/Df(3 L) and *abp1* ^WH f05024^ larvae were specifically caused by *abp1* disruption.

### Presynaptic Abp1 is critical for neuromuscular bouton formation

In order to dissect whether Abp1's critical role in synapse formation and in nerve terminal branching relies on pre- or postsynaptic functions or both, we next reexpressed Abp1 in the *abp1* ^KO^/Df(3 L) background. Exclusive expression of Abp1 at the muscle side was not sufficient to restore normal type Ib synaptic bouton formation during NMJ development. The *abp1* knock-out phenotype fully persisted upon C57-*Gal4*-driven expression at the postsynaptic side (Fig. 4D,E).

In contrast, motoneuronal reexpression of Abp1 using OK371-*Gal4* (see Fig. S1 for driver control) completely rescued the impairment in synaptic bouton formation (Fig. 4D,E). This confirmed that the observed defects in synaptic bouton formation observed in *abp1* ^KO^/Df(3 L) flies are specifically due to ablation of *abp1*. Furthermore, the rescue experiments revealed that loss of presynaptic Abp1 functions accounts for the reduction in type Ib bouton number seen in *abp1* knock-out flies.

### Nerve terminal branching seems to be secured by crosstalk between pre- and postsynaptic compartments

The impairments of axon terminal branching observed upon *abp1* knock-out were also fully rescued by reexpression of Abp1 in motoneurons of *abp1* ^KO^/Df(3 L) flies using OK371-*Gal4* (Fig. 4D,F) These data showed that presynaptic Abp1 is sufficient for proper nerve terminal branching.

Intriguingly, however, muscle-specific Abp1 reexpression driven by C57-*Gal4* also restored proper axonal branching to *wt* levels (Fig. 4D,F). The number of axonal branching points in NMJs of *abp1* ^KO^/Df(3 L) flies with postsynaptically reexpressed Abp1 was indistinguishable from that of *wt* flies. These observations suggest that nerve terminal branching is secured by some bidirectional crosstalk between pre- and postsynaptic entities.

### High resolution confocal and STED microscopical analyses of Abp1 localization in the presynaptic compartment

**Figure 5:**
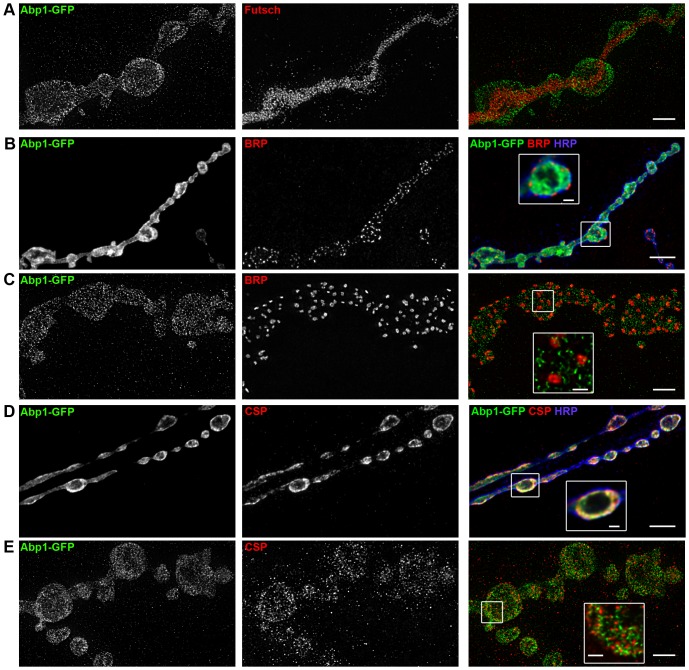
Super-resolution 2-channel STED microscopy shows distinct localization of Abp1-GFP in presynaptic nerve terminals at NMJs. Maximum intensity projections of deconvolved confocal (**B,D**) as well as STED images (**A,C,F**) recorded from NMJs at muscle 6/7 (A2). (**A**) STED images of Abp1-GFP and Futsch (outlining presynaptic microtubules) show no colocalization between the two proteins. Abp1 often displays enrichments at the cell cortex. Bar, 2 µm. (**B**) Confocal imaging of Bruchpilot (BRP) and HRP shows the localization of Abp1-GFP near the plasma membrane but not in the middle of boutons. There is no overlap with BRP marking the active zone core structure, as seen in the merge and in the magnified inset representing as single image plane. Bar, 5 µm; bar in inset, 1 µm. (**C**) STED microscopy confirms the lack of spatial overlap of Abp1 and BRP. Bars, 2 µm; bar in insert (single slice image), 500 nm. (**D**) Confocal microscopy of immunostainings for CSP suggests a strong colocalization of Abp1-GFP with CSP. Bar, 5 µm; in inset (single image plane), 1 µm. (**E**) However, 2-channel STED microscopy shows no colocalization of Abp1 with CSP but Abp1 rather localizes adjacent to individual anti-CSP-stained synaptic vesicles. Bars, 2 µm; bar in inset (single image plane), 500 nm.

Mammalian Abp1 has been reported to interact with the active zone protein Piccolo [24] and with the synaptic vesicle protein synapsin I, which helps to tether synaptic vesicles to actin filaments [25]. In order to improve the signal-to-noise ratio and to make sure that only presynaptic Abp1 is analyzed, we expressed Abp1-GFP by OK371-*Gal4* to specifically analyze the localization of Abp1 in the presynaptic compartment of NMJs at high resolution. STED microscopy of NMJs of OK371-*Gal4*+Abp1-GFP flies revealed that Abp1 is predominantly localized near the plasma membrane of presynaptic boutons and much less in the internal, microtubuli-enriched areas of the nerve terminal marked by Futsch (Fig. 5A).

Coimmunolabeling of Bruchpilot (BRP), a structural component of active zones of synaptic vesicle fusion in flies [26], showed by both confocal as well as by STED microscopy that Abp1 is not spatially overlapping with the machinery for synaptic vesicle exocytosis. Abp1 and Bruchpilot localizations were often very close to each other but always were clearly segregated (Fig. 5B,C).

Further examinations using CSP as a marker for synaptic vesicles [27] showed that Abp1 displayed a striking spatial overlap with synaptic vesicle pools at the resolution of confocal microscopy (Fig. 5D). However, when this localization of Abp1 close to the cell cortex was followed up at higher resolution using STED microscopy, it became apparent that the Abp1 and the CSP localization are usually not superposable but that both proteins actually reside directly adjacent to each other. While CSP immunosignals hereby were regular in size, as it can be expected from single synaptic vesicles, the Abp1 immunostaining adjacent to synaptic vesicles was more irregular in shape (Fig. 5E).

The STED microscopy analyses thus suggest that rather than functions directly at the active zones or at synaptic vesicles, the critical role of Abp1 in NMJ development relates to actin cytoskeletal functions inbetween vesicles and/or at the presynaptic bouton membrane cortex.

### Abp1 interacts with both WASP and Scar in the fly nervous system

**Figure 6:**
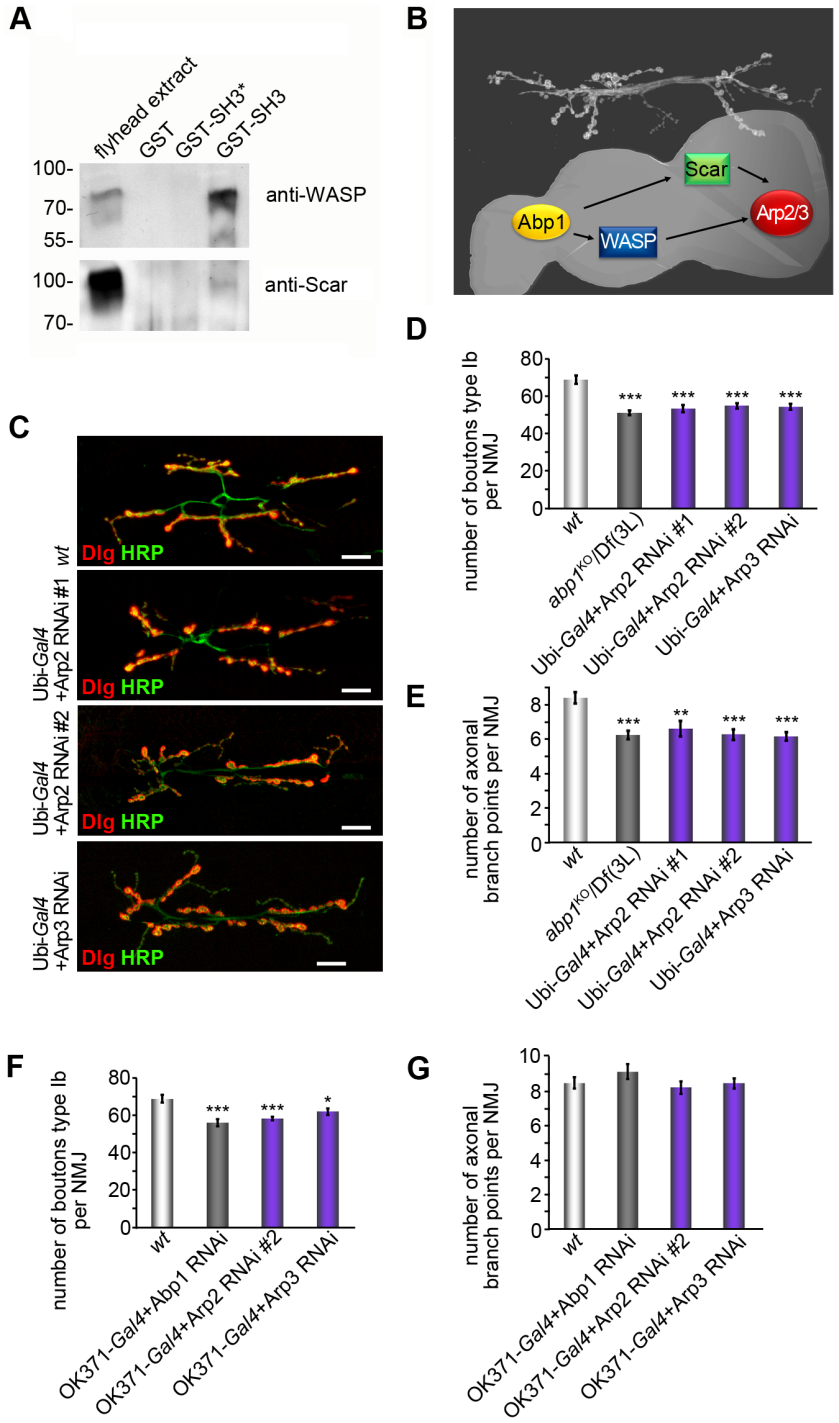
Arp2 and Arp3 loss-of-function mirror the defects in NMJ development observed upon *abp1* deficiency. (**A**) The immobilized SH3 domain of Abp1 (GST-SH3) specifically precipitates endogenous WASP and Scar from fly head extracts whereas GST and a point-mutated SH3 domain (GST-SH3*; P523A mutation), respectively, do not. (**B**) Model depicting that Abp1 interfaces with both WASP and Scar and may regulate Arp2/3 complex dependent actin dynamics at the NMJ using both Arp2/3 complex activation pathways. (**C**) NMJs at muscles 6/7 of abdominal segment A2 of *wt*, Arp2 RNAi #1, Arp2 RNAi #2 and Arp3 RNAi expressing larvae stained with HRP (green) and anti-Dlg (red). (**D,E**) Quantitative analyses of type Ib boutons and of terminal axonal branch points visualize that Arp2/3 complex deficiency phenocopies *abp1* knock-out in reducing type Ib bouton number and terminal branch points of the axon within NMJs. (**F,G**) Quantitative analyses revealing that larvae with presynaptic Arp2 RNAi #2 or Arp3 RNAi phenotypically mirror presynaptic Abp1 RNAi, as specifically type Ib bouton formation but not the formation of terminal axonal branch points is affected. Data represent mean±SEM. n = 18–36/genotype. * = p<0.05, ** = p<0.01 and *** = p<0.001. One way ANOVA post Tukey.

Abp1 has been demonstrated to control the activity of the Arp2/3 complex activator N-WASP by releasing its autoinhibition in mammals [18] and to steer Scar functions in bristle formation in flies [15]. We therefore addressed whether Abp1 would also interact with Scar in the nervous system and whether fly Abp1 would additionally interact with the only expressed relative of N-WASP in flies, i.e. with WASP. Protein interaction studies using immobilized, purified, recombinant GST-Abp1 SH3 domain fusion proteins demonstrated that both endogenous WASP and Scar were specifically precipitated from fly head extracts. The interactions were brought about by the classical SH3 domain/PxxP interaction interface, as mutating Abp1's SH3 domain accordingly (P523A) disrupted both Scar and WASP interaction (Fig. 6A).

Immunolocalization analyses of endogenous WASP and Scar suggest that similar to Abp1 also these two Arp2/3 complex activators are localized to both sides of the NMJ and show considerable overlap with their binding partner Abp1 (Fig. S2A–D).

Since Abp1 interacts with both known Arp2/3 complex activators, it may modulate Arp2/3 complex functions by both the Scar and the WASP pathway (Fig. 6B). As the published analyses of HRP-labeled boutons conducted for some of the established WASP or Scar pathway mutants were hard to compare to our quantitative evaluations of Dlg-positive type Ib boutons and since branching of axonal terminals to our knowledge has not been addressed for any WASP or Scar pathway mutants, we evaluated both parameters for several WASP and Scar loss-of-function conditions. Summarized, we did not observe any consistent, statistical significant, inhibitory effects on formation of type Ib boutons or on branching of axonal terminals in heterozygous *scar* ^Δ37/+^, *scar* ^k03107/+^ mutants, heteroallelic *wasp* ^1^/wasp^3^ mutants and in WASP and Scar RNAi flies, respectively (Fig. S2E–J).

### Arp2/3 loss-of-function mirrors Abp1 knock-out and RNAi

Abp1 associates with both Scar and WASP and spatially overlaps with both of these Arp2/3 complex activators at the NMJ (Fig. S2A–D). Yet, neither loss of WASP nor reduction of Scar consistently phenocopied *abp1* knock-out (Fig. S2E–J). We therefore speculated that the WASP and Scar pathways of Arp2/3 complex activation may show some functional redundancy in NMJ development. As both pathways converge onto the same molecular component down-stream of WASP and Scar, the Arp2/3 complex (Fig. 6B), such a model would demand that ubiquitous knock-down of Arp2/3 complex components should mirror the defects observed upon *abp1* knock-out. RNAi-mediated knock-down of Arp2 in two different strains as well as knock-down of Arp3 indeed all consistently led to defects in NMJ development (see Fig. S1 for driver control for phenotypes) that were similar to those observed upon *abp1* knock-out, as also upon Arp2/3 complex loss-of-function type Ib bouton numbers were significantly reduced (Fig. 6C,D; also compare Fig. 4A,B for *abp1* knock-out phenotype).

The branching of the axon terminals also was reduced upon Arp2 RNAi and Arp3 RNAi, respectively, when compared to *wt* (Fig. 6C,E). Again, these Arp2 and Arp3 loss-of-function phenotypes were qualitatively and quantitatively similar to that observed in *abp1* ^KO^/Df(3 L) flies (−25%) (Fig. 4A,C). Arp2 and Arp3 loss-of-function therefore very closely mirrored both defects of NMJ formation observed upon *abp1* knock-out.

In line with the results from our rescue experiments, presynaptic Abp1 RNAi (using the motoneuronal driver OK371) demonstrated that presynaptic Abp1 is crucial for the formation of type Ib synaptic boutons but alone has no significant impact on the branching of nerve terminals (Fig. 6F,G).

Presynaptic knock-down of the Arp2/3 complex components Arp2 and Arp3, respectively, using the same driver also led to a reduction of type Ib synaptic bouton numbers but not of branching points of the nerve terminals (Fig. 6F,G). Thus, the specific, presynaptic importance of Abp1 for proper NMJ development is mirrored by a similar presynaptic requirement for the Arp2/3 complex for type Ib synaptic bouton formation.

### Presynaptic Abp1-mediated functions in the formation of type Ib synaptic boutons as well as in branching of axon terminals critically depend on the Arp2/3 complex

**Figure 7:**
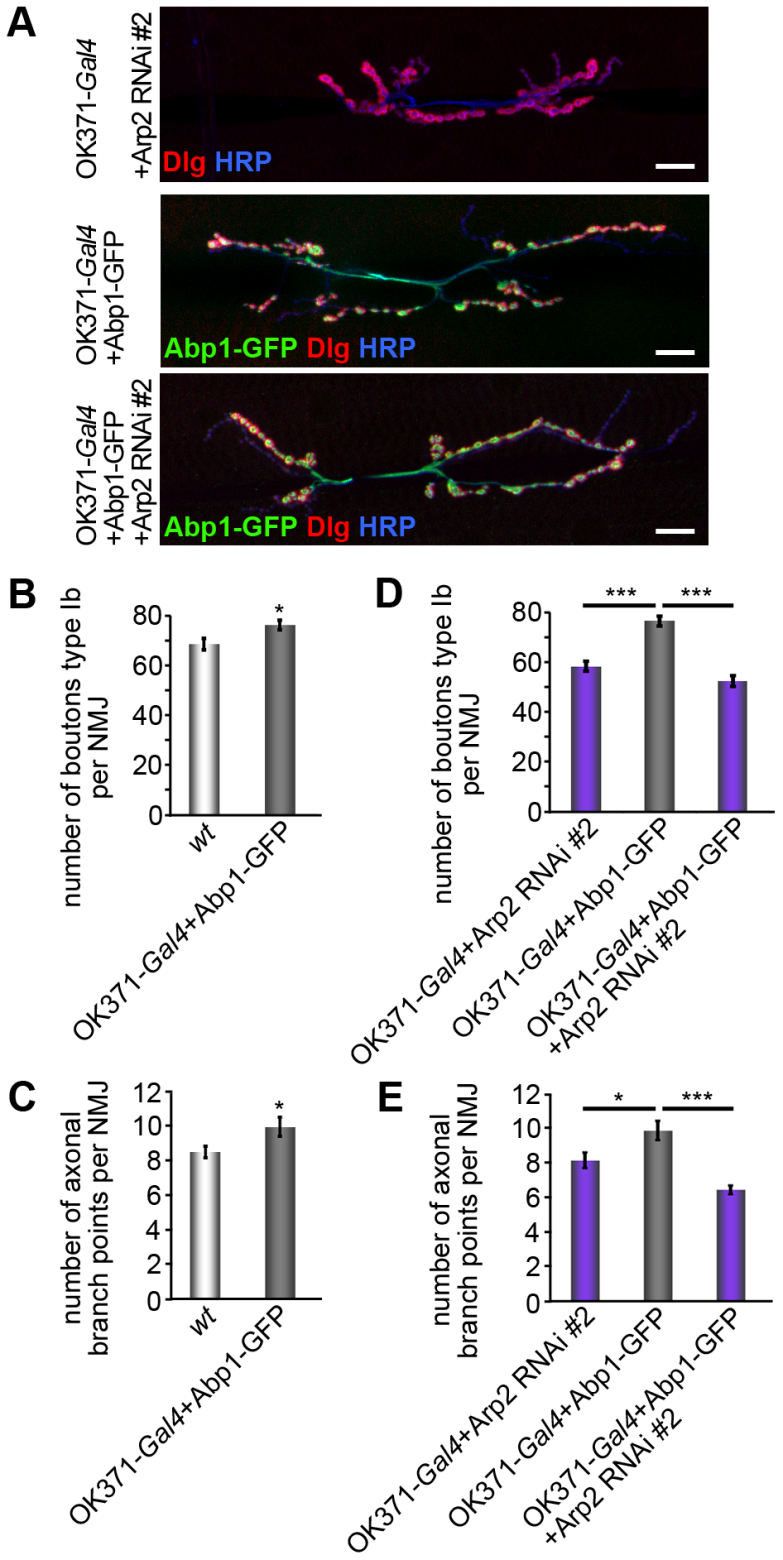
Presynaptic Abp1-dependent functions in NMJ development critically rely on presynaptic Arp2/3 complex. (**A**) Anti-GFP, HRP and anti-Dlg-stained NMJs at muscles 6/7 of abdominal segment A2 from larvae expressing OK371-*Gal4*+Arp2 RNAi #2, OK371-*Gal4*+Abp1-GFP and OK371-*Gal4*+Abp1-GFP+Arp2 RNAi #2, respectively. Bar, 20 µm. (**B–E**) Quantitative analyses of type Ib boutons and of nerve terminal branch points show that the presynaptic expression of Abp1-GFP leads to both an increase in bouton number (**B**) and to an increase in terminal axonal branch points (**C**), i.e. to phenotypes opposite to Abp1 knock-out. (**D,E**) Presynaptic knock-down of Arp2 suppresses both of the Abp1-induced effects. Abp1's functions in the presynapse of NMJs thus are critically dependent on presynaptic Arp2/3 complex functions. Data represent mean±SEM. n = 20–22/genotype. * = p<0.05 and *** = p<0.001. Student's t-test (**B,C**) and One way ANOVA post Tukey (**D,E**).

In order to directly address a putative requirement of the Arp2/3 complex for Abp1 functions, we next analyzed whether an excess of presynaptic Abp1 has any effects on NMJ expansion. OK371-*Gal4*+Abp1-GFP flies displayed a significant increase in type Ib boutons when compared to OK371-*Gal4* controls and *wt* flies, respectively (Fig. 7A,B; Fig. S1). Also terminal axonal branch points were increased significantly (Fig. 7A,C). Presynaptic excess of Abp1 thus had effects opposite to Abp1 knock-out in NMJ formation.

These observations prompted us to address the hypothesized critical role of the Arp2/3 complex for Abp1-mediated functions in the nerve terminal by knocking down Arp2 presynaptically in flies with a presynaptic excess of Abp1. Indeed, when Abp1-GFP was expressed together with an Arp2 RNAi, the increases of the numbers of type Ib boutons and of branching points induced by the presynaptic excess of Abp1 both were completely suppressed (Fig. 7A,D,E). Thus, Abp1's functions in the development of NMJs critically depend on the Arp2/3 complex.

### Arp2/3 complex loss-of-function phenocopies *abp1* deficiency in larval migration

**Figure 8:**
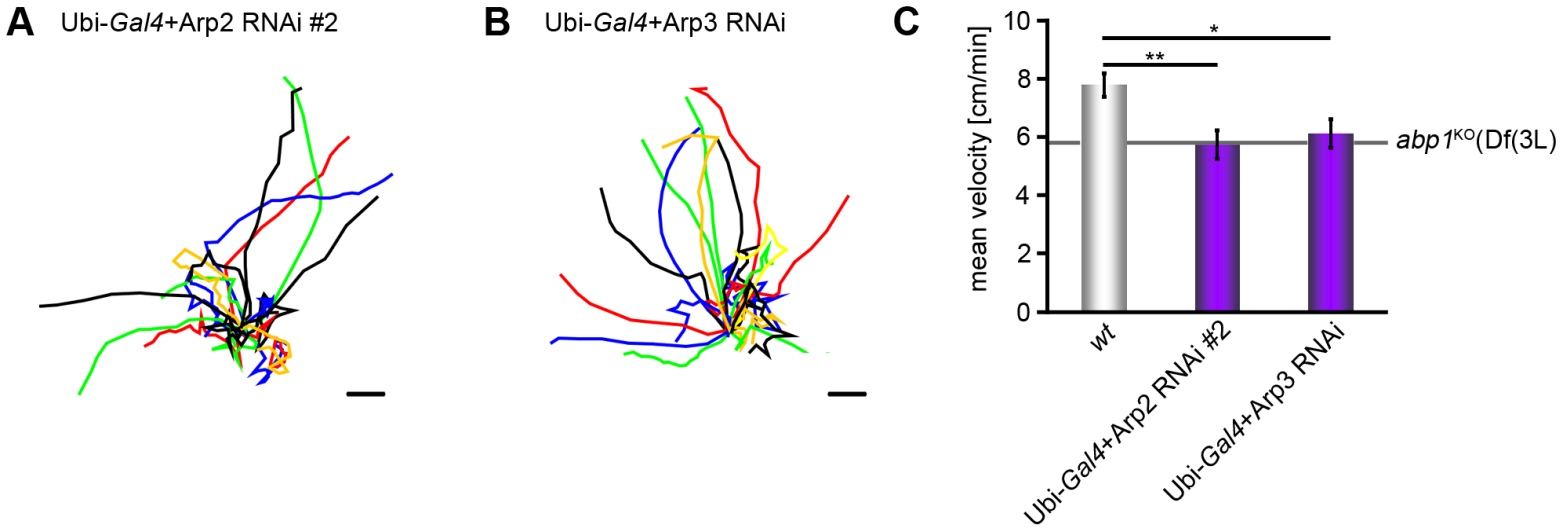
Knock-down of Arp2 and Arp3 lead to larval migration defects phenocopying *abp1* knock-out. (**A,B**) Summarized migration tracks with centered common start points (bars, 5 mm) of Ubi-*Gal4*+Arp2 RNAi #2 (**A**) and Ubi-*Gal4*+Arp3 RNAi (**B**). (**C**) Mean velocities of larval migration (n = 19–20). Data represent mean±SEM. * = p<0.05 and ** = p<0.01. One way ANOVA post Tukey.

Our examinations revealed that Abp1 and the Arp2/3 complex work together closely in proper NMJ development. Knock-out of *abp1* also resulted in a reduced larval migration phenotype (Fig. 1). Thus, we finally asked whether RNAi against Arp2/3 complex components would lead to similar impairments. As in NMJ development, Arp2 and Arp3 RNAi gave consistent results (for driver control see Fig. S3) and resulted in shortened larval tracks in larval migration assays (Fig. 8A,B).

Quantitative analyses of Arp2 and Arp3 RNAi flies unraveled that Arp2/3 complex depletion leads to reduced larval migration velocities that were phenocopying the defects observed upon *abp1* knock-out (Fig. 8C).

## Discussion

Proper formation of neuronal connectivity within the central nervous system and in the periphery requires that axon terminals form synapses and that axon terminals expand the presynaptic, signal-sending compartment by branching. Our analyses using an established UAS-Arp2 RNAi line [28] and two further lines with suppressed Arp2/3 complex functions demonstrate that Arp2/3 complex-mediated actin cytoskeletal dynamics is critically involved in both synaptic bouton formation and axon terminal branching. Knock-out of the *abp1* gene revealed that also Abp1 is a critical factor in both aspects of NMJ development. Thus, intriguingly, with Abp1 and the Arp2/3 complex both synaptic bouton formation and axon terminal branching rely on the same molecular components and these molecular components both represent mechanisms of actin dynamics.

The specificity of the observed defects at *abp1* mutant NMJs is demonstrated by the facts that (i) various allelic combinations displayed virtually identical phenotypes, (ii) *wt* situations in both bouton formation and axon terminal branching were restored by precise excision of the PBac insertion WH f05024 and iii) expression of the F-actin binding protein Abp1 in *abp1* ^KO^/Df(3 L) backgrounds fully suppressed the *abp1* loss-of-function phenotypes in both processes.

Similar to general axon development, the expansion of axon terminals at *Drosophila* larval NMJs is thought to be primarily driven by the microtubular cytoskeleton [3], [29]. Our findings add to the idea that the actin cytoskeleton also is required for controlling bouton formation and axon terminal branching. In support of our finding that functions of the actin nucleator Arp2/3 complex and Abp1 are crucial for proper NMJ formation, two proteins that mediate crosstalk between microtubules and actin filaments, Diaphanous and Baz/Par-3, were implicated in NMJ morphology control in flies and worms, respectively [8], [30]. NMJ expansion and terminal axon branching in *Drosophila* are furthermore both promoted by PI3K signaling [11]–[13], which for example controls actin cytoskeletal dynamics via PIP_3_ levels. Moreover, it has been reported that fly NMJ expansion is controlled by Rac, a principal regulator of F-actin dynamics [7]. In mammals, Abp1 is a widely expressed, Rac1-responsive cytoskeletal component [14] that associates with dynamic F-actin structures at the cell cortex [14], [23], [25], [31]. *Drosophila* Abp1 has recently been revealed to associate with membranes containing the PI3K reaction product PIP_3_ and to localize to F-actin-rich lamellipodia [15].

Our expression analyses showed that fly Abp1, similar to its mammalian relative [14], shows moderate expression in muscles but is highly expressed in the CNS. The rescue experiments we conducted showed that reexpression of Abp1 at the neuronal side restores proper synaptic bouton formation and axon terminal branching in *abp1* mutants. Motoneuronal expression of Abp1 RNAi corroborated the importance of presynaptic Abp1 in type Ib synaptic bouton formation. An excess of Abp1 in motoneurons led to phenotypes opposite to *abp1* knock-out, both the number of type Ib synaptic boutons as well as the number of branch points in the axon terminals were significantly increased.

Besides its cytoskeletal functions, Abp1 has been implicated in receptor-mediated endocytosis via interactions with dynamin [25], [32], [33]. In flies, disturbed BMP receptor endocytosis causes excessive BMP signaling leading to NMJ overgrowth [34]–[35]. At larval NMJs several endocytosis mutants have also been associated with an excess of so-called satellite boutons [34]. In contrast, *abp1* mutants display smaller and less complex NMJs with fewer type Ib synaptic boutons and reduced branching of nerve terminals. The role of Abp1 in NMJ development thus does not seem related to endocytosis. Rather, the fact that depletion of the integral Arp2/3 complex components Arp2 and Arp3 led to impairments in NMJ formation that were indistinguishable from *abp1* loss-of-function phenotypes strongly suggests that the impaired synaptic bouton formation and the impaired axon terminal branching both reflect a loss of actin cytoskeletal functions during NMJ development. In line with this, the complete suppression of the Abp1-mediated type Ib synaptic bouton formation and branching of nerve terminals by concomitantly knocking down Arp2/3 complex functions revealed that Abp1 functions in the NMJ are fully dependent on the Arp2/3 complex.

Little is known about the role of Abp1 in the formation or structural organization of presynaptic terminals of vertebrate neurons. The identification of the vertebrate-specific presynaptic active zone scaffold protein Piccolo as binding partner of Abp1 [24] suggested yet to be deciphered roles of Abp1 in presynaptic organization in vertebrates. It is conceivable that such a presynaptic function of Abp1 in vertebrates will also involve the Arp2/3 complex similar to the data we obtained for presynapse formation in *Drosophila*. Our STED microscopy analyses suggest that this critical role of Abp1 in presynaptic terminals is not directly linked to the core of the active zone and the exocytic fusion of synaptic vesicles at the active zone as neither colocalization with Bruchpilot nor with CSP was observed at the high resolutions achievable by STED microscopy. Abp1 functions in presynaptic boutons may thus rather relate to control of actin dynamics involved in modulating topology and dynamics of the presynaptic plasma membrane.

Several observations are in line with the intimate functional relationship between Abp1 and Arp2/3 complex-mediated actin nucleation, which we here unraveled in NMJ development. In rat hippocampal neurons, Abp1 was shown to support the maturation and head expansion of dendritic spines. This involved F-actin and ProSAP/Shank scaffold protein interactions and the Arp2/3 complex [31]. Upon Abp1 SH3 domain-mediated association, vertebrate Abp1 releases the autoinhibition of the Arp2/3 complex activator N-WASP and was shown to thereby steer Arp2/3 complex functions in early morphogenesis of hippocampal neurons [18]. Fly Abp1 employs the Arp2/3 complex in bristle formation via the Arp2/3 complex activator Scar [15].

Our finding that Abp1 and Arp2/3 complex-mediated functions are critical for proper NMJ formation furthermore is in line with studies in mammalian cells showing that F-actin disruption during synaptogenesis results in fewer presynapses [36]–[39] and by recent observations, which identified actin filament-promoting proteins such as lamellipodin to be critical for both synapse and axonal branch formation in the AIY interneuron of *C. elegans* [10].

Seeming discrepancies arise from reports describing no or even positive effects of impairing Arp2/3 complex pathway components on some NMJ expansion parameters. *Wasp* mutants were reported to show an increased overall NMJ length [4], [40] and an excess of boutons [4]. Similar results were reported upon postsynaptic Arp2 and WASP RNAi, whereas presynaptic Arp2 RNAi effects using the pan-neuronal C155-*Gal4* as a presynaptic driver. We used the very effective and motoneuron-specific OK371-*Gal4* to drive our different Arp2 and Arp3 RNAis and evaluated parameters different from the above studies, which were based on anti-HRP stainings of boutons overall and did not specifically focus on type Ib boutons. The branch points of the axon terminals were not analyzed in any of the above mentioned studies. Thus, much of the seemingly discrepancy in the Arp2/3 complex and WASP literature may in fact be due to different parameters being evaluated, caused by different RNAi and mutants being used and/or depend on pre- and postsynaptic as well as ubiquitous impairments of NMJ functions.

The same seems true for the analyses of Scar and Scar complex components, which additionally are hampered by the fact that homozygous *scar* knock-out flies not viable [41] and the stability of Scar complex components depend on each other. Studies on Scar and Scar complex components are not fully consistent and focused on overall NMJ length and the appearance of small, Dlg-negative satellite boutons [42]–[44] rather than on type Ib bouton formation and branching of axon terminals. We observed that Scar loss-of-function NMJs were mostly not significantly different from *wt* NMJs and that *scar^Δ37^*/+ and *scar^k03107^*/*+* flies yielded somewhat inconsistent trends in both NMJ developmental parameters we examined.

Our observation that neither disrupting *scar* nor *wasp* functions individually led to consistent impairments of type Ib synaptic bouton formation and/or terminal axon branching may either be explained by a dependence of proper NMJ development on alternative Arp2/3 complex activators, i.e. on WASH and WHAMY, which in part, however seems not to be in line with their differential expression patterns [45], or by some redundancy of WASP and Scar-mediated Arp2/3 complex activation pathways in NMJ formation, which may be reflected by our finding that Abp1 does not only interact with Scar but also with WASP in the fly nervous system.

The surprising observation that the impairment of terminal branching was restored by pre- or postsynaptic expression of Abp1 suggests that nerve terminal branching is secured by bidirectional crosstalk between pre- and postsynaptic cytoskeletal arrangements. The finding that restoring Abp1 functions at either side of the NMJ is sufficient to bring about a normal extent of axon terminal branching in NMJ development may also help to explain how axon branching on the one hand can precede synapse formation, yet, on the other also occurs after synapse formation in a manner coordinated with synapses that had been formed.

As expected, also larval migration obviously was mechanistically more complexly relying on Abp1 and Arp2/3 complex functions than type Ib bouton formation. Larval migration was not rescued to *wt* levels by Abp1 reexpression in muscles or motoneurons (Fig. S4). This suggests that larval migration is affected by impairments beyond the NMJ defects we unraveled. This likely includes additional defects in sensory performances and/or in information processing in the brain, which may also be affected by knock-out of *abp1*. Indeed, studies in mammalia suggest that Abp1 is a crucial player in presynaptic transmission [33], neuromorphogenesis [16] and postsynaptic organization [23], [31] of hippocampal neurons and Purkinje cells and examinations in flies additionally revealed that *abp1* knock-out impacts on sensory systems, as compound eyes of abp1 knock-out flies show phenotypes representing a mixture of WASP and Scar mutant phenotypes (our unpublished data) and bristles of a*bp1* knock-out flies show a disrupted cytoskeletal organization that interestingly also closely relates to Arp2/3 complex loss-of-function phenotypes [15].

Taken together, our study reveals an intimate functional relationship of Abp1 and the Arp2/3 complex, as first, Abp1 physically binds to Arp2/3 complex activators, second, both Abp1 and the Arp2/3 complex loss-of-function caused similar phenotypes in both larval migration and NMJ development, third, both cytoskeletal components were specifically crucial in the presynaptic compartment and, fourth, we were able to directly demonstrate that Abp1-mediated functions in the presynapse are dependent on functions of the actin nucleator Arp2/3 complex. Our data thus strongly suggest that with Abp1 and Arp2/3 complex-mediated actin nucleation both synapse formation and terminal axonal branching share common critical components and molecular mechanisms.

## Supporting Information

Figure S1 **NMJs of driver strains.** (**A,B**) Quantification of type Ib bouton numbers (**A**) and terminal axonal branch points (**B**) of *wt* and OK371-*Gal4* and Ubi-*Gal4* expressing larvae. One way ANOVA post Tukey.
(TIF)

Figure S2 **Colocalization and genetic interaction analyses of** ***abp1*** **with** ***wasp*** **and** ***scar*** **in NMJ development.** (**A–D**) Confocal images of immunostained 3^rd^ instar larval NMJs show that Abp1 partially colocalizes with both Scar and WASP at NMJs. (**B,D**) Quantitative analyses of fluorescence intensity plots along the lines shown in **A** and **C** allow for resolving spatially distinct fluorescence maxima for Scar and WASP (marked by arrow heads in **B** and **D**). Anti-Scar and anti-WASP immunostaining spans the localization of both Abp1 and Dlg. (**E**) NMJs at muscles 6/7 of abdominal segment A2 analyzed by presynaptic (HRP) and postsynaptic markers (Dlg). Shown are examples of NMJs from *wasp* ^1/3^and *abp1* ^KO^/Df(3L)*+wasp* ^1/3^. Bar, 20 µm. (**F,G**) Quantitative analyses of type-1b bouton numbers and NMJ branch points of the different *wasp*-deficient flies as well as pre- and postsynaptic expression of Wasp RNAi in comparison to *wt* and *abp1* knock-out demonstrate that there is no significant difference between *wt* and *wasp*-deficient strains. (**H**) HRP and anti-Dlg-stained NMJs of heterozygous *scar* ^k03107^ and *abp1* ^KO^/Df(3L)+*scar* ^k03107/+^ larvae. Bar, 20 µm. (**I,J**) Quantitative analyses of type-Ib boutons (**I**) and of nerve terminal branch points (**J**) of *scar*-deficient strains show that only a postsynaptic reduction of Scar using C57-*Gal4*+Scar RNAi flies leads to a reduction of bouton number similar to the *abp1* knock-out phenotype.
(TIF)

Figure S3 **Larval migration of driver strain Ubi-** ***Gal4*** **compared to** ***wt*** **.** Mean velocities of larval migration (n = 38 and 11, respectively). Data represent mean±SEM. Student's t-test.
(TIF)

Figure S4 **Pre- and postsynaptic reexpression of Abp1 in** ***abp1*** **knock-out flies does not restore larval migration.** (**A,B**) Summarized migration tracks with centered common start points (bars, 5 mm) of C57-*Gal4*+Abp1+*abp1* ^KO^/Df(3L) (**A**) and OK371-*Gal4*+Abp1+*abp1* ^KO^/Df(3L)flies (**B**). (**C**) Mean velocities of larval migration (n = 19–21). Note that reexpression of Abp1 in only motoneurons and muscles, respectively, did not cause significant differences in larval migration when compared to *abp1* knock-out. Data represent mean±SEM. * = p<0.05 and ** = p<0.01. One way ANOVA post Tukey.
(TIF)
