# Supplementary figures and images for: Terminal Axonal Arborization and Synaptic Bouton Formation Critically Rely on Abp1 and the Arp2/3 Complex
Source: PLoS One. 2014 May 19;9(5):e97692. doi: 10.1371/journal.pone.0097692 (PMC4026379; doi:10.1371/journal.pone.0097692)

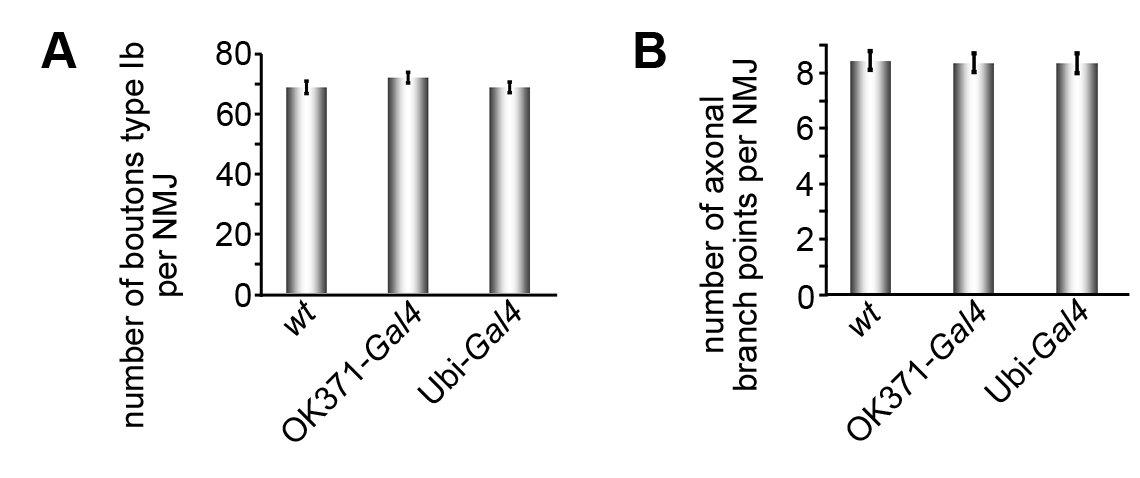

Supplement: Figure S1 — NMJs of driver strains. (A,B) Quantification of type Ib bouton numbers (A) and terminal axonal branch points (B) of wt and OK371-Gal4 and Ubi-Gal4 expressing larvae. One way ANOVA post Tukey. (TIF) [file pone.0097692.s001.tif]

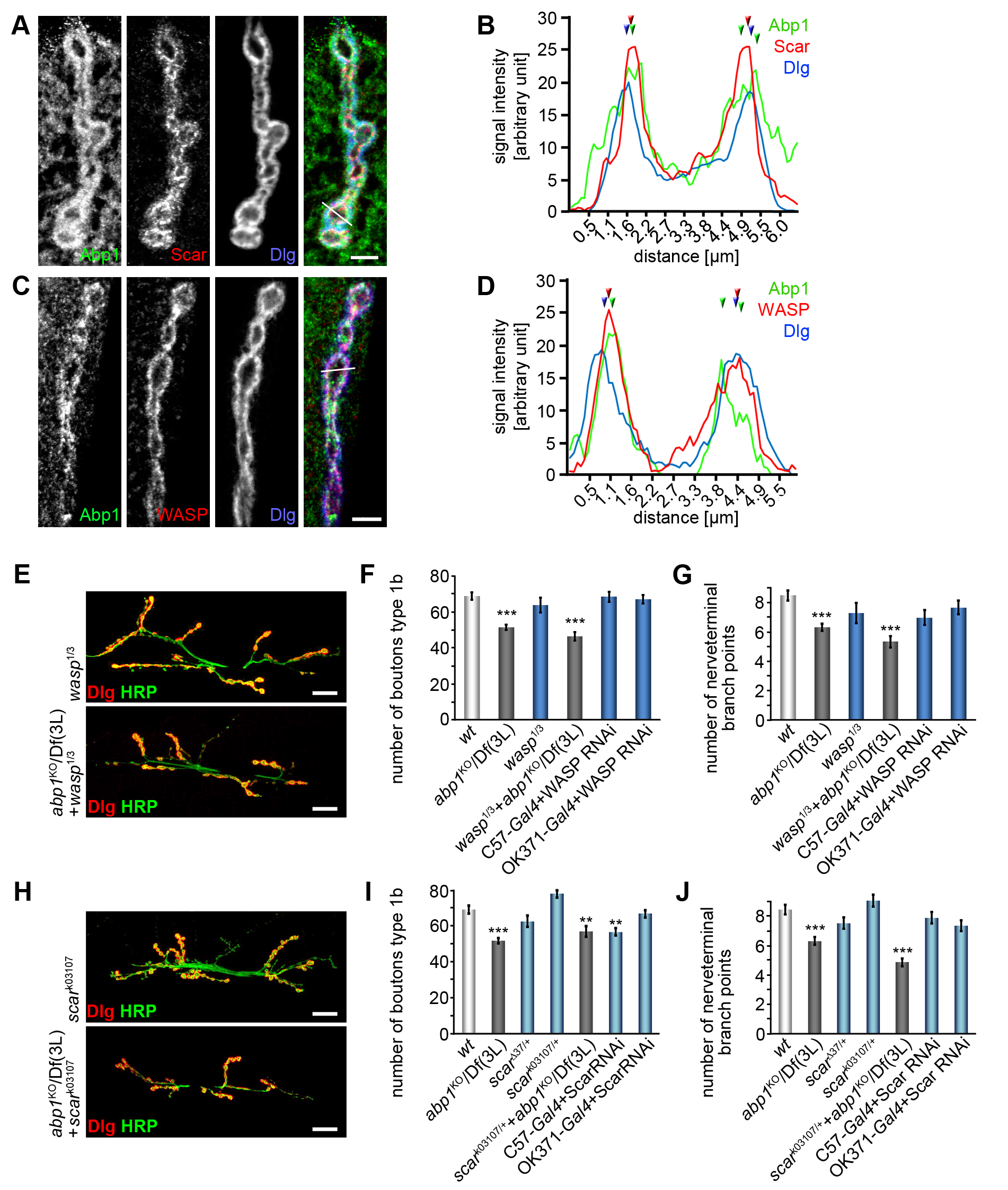

Supplement: Figure S2 — Colocalization and genetic interaction analyses of abp1 with wasp and scar in NMJ development. (A–D) Confocal images of immunostained 3rd instar larval NMJs show that Abp1 partially colocalizes with both Scar and WASP at NMJs. (B,D) Quantitative analyses of fluorescence intensity plots along the lines shown in A and C allow for resolving spatially distinct fluorescence maxima for Scar and WASP (marked by arrow heads in B and D). Anti-Scar and anti-WASP immunostaining spans the localization of both Abp1 and Dlg. (E) NMJs at muscles 6/7 of abdominal segment A2 analyzed by presynaptic (HRP) and postsynaptic markers (Dlg). Shown are examples of NMJs from wasp 1/3and abp1 KO/Df(3L)+wasp 1/3. Bar, 20 µm. (F,G) Quantitative analyses of type-1b bouton numbers and NMJ branch points of the different wasp-deficient flies as well as pre- and postsynaptic expression of Wasp RNAi in comparison to wt and abp1 knock-out demonstrate that there is no significant difference between wt and wasp-deficient strains. (H) HRP and anti-Dlg-stained NMJs of heterozygous scar k03107 and abp1 KO/Df(3L)+scar k03107/+ larvae. Bar, 20 µm. (I,J) Quantitative analyses of type-Ib boutons (I) and of nerve terminal branch points (J) of scar-deficient strains show that only a postsynaptic reduction of Scar using C57-Gal4+Scar RNAi flies leads to a reduction of bouton number similar to the abp1 knock-out phenotype. (TIF) [file pone.0097692.s002.tif]

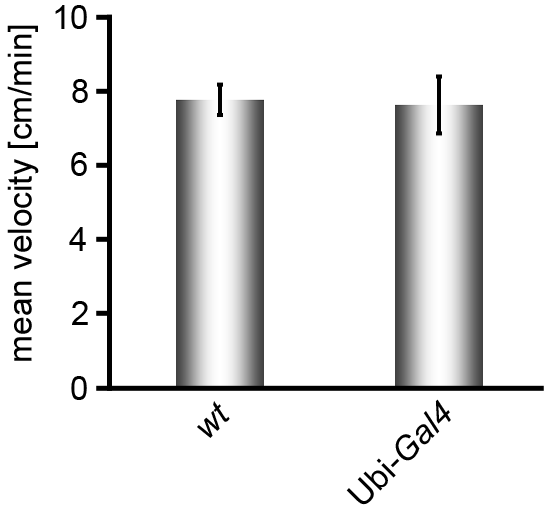

Supplement: Figure S3 — Larval migration of driver strain Ubi- Gal4 compared to wt . Mean velocities of larval migration (n = 38 and 11, respectively). Data represent mean±SEM. Student's t-test. (TIF) [file pone.0097692.s003.tif]

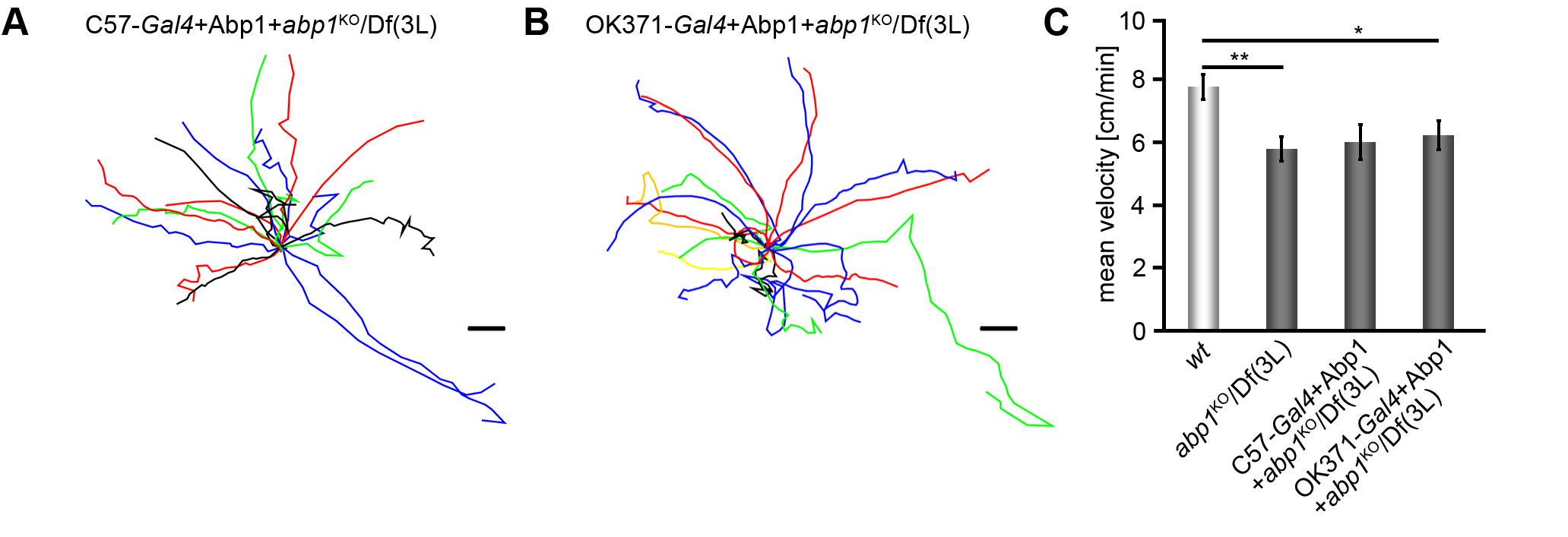

Supplement: Figure S4 — Pre- and postsynaptic reexpression of Abp1 in abp1 knock-out flies does not restore larval migration. (A,B) Summarized migration tracks with centered common start points (bars, 5 mm) of C57-Gal4+Abp1+abp1 KO/Df(3L) (A) and OK371-Gal4+Abp1+abp1 KO/Df(3L)flies (B). (C) Mean velocities of larval migration (n = 19–21). Note that reexpression of Abp1 in only motoneurons and muscles, respectively, did not cause significant differences in larval migration when compared to abp1 knock-out. Data represent mean±SEM. * = p<0.05 and ** = p<0.01. One way ANOVA post Tukey. (TIF) [file pone.0097692.s004.tif]
